# Formation of open ruthenium branched structures with highly exposed active sites for oxygen evolution reaction electrocatalysis†

**DOI:** 10.1039/d5sc01861g

**Published:** 2025-04-16

**Authors:** Sa Xiao, Yuhan Xie, Agus R. Poerwoprajitno, Lucy Gloag, Qinyu Li, Soshan Cheong, Zeno R. Ramadhan, Ingemar Persson, Yoshiki Soda, Dale L. Huber, Liming Dai, J. Justin Gooding, Richard D. Tilley

**Affiliations:** a School of Chemistry, The University of New South Wales Sydney NSW 2052 Australia justin.gooding@unsw.edu.au r.tilley@unsw.edu.au; b Center for Integrated Nanotechnologies, Sandia National Laboratories Albuquerque NM 87185 USA; c Research School of Chemistry, The Australian National University Canberra ACT 2601 Australia; d Mark Wainwright Analytical Centre, The University of New South Wales Sydney NSW 2052 Australia; e School of Chemical Engineering, The University of New South Wales Sydney NSW 2052 Australia; f Australian Centre for NanoMedicine, The University of New South Wales Sydney NSW 2052 Australia

## Abstract

The formation of exposed active sites that have high activity and stability for oxygen evolution reaction (OER) catalysis is a significant opportunity for improving water electrolysers. Low-index facets surface Ru can achieve both high activity and stability for OER. Here, we present a new catalyst design where low-index faceted Ru branches are grown off the corners of Pt nanocubes, forming open Ru branched nanoparticles. This open branched structure, exposing low-index facets on its length-tunable branch, enables a high electrochemically active surface area (ECSA), achieving high activity and stability for OER. This design strategy and synthetic control provide a principle for achieving high-performance OER nanocatalysts.

## Abstract 2

The open Ru branched nanostructures provide high accessibility to incoming reagents and high electrochemically active surface areas for catalysing the oxygen evolution reaction.

## Graphical abstract

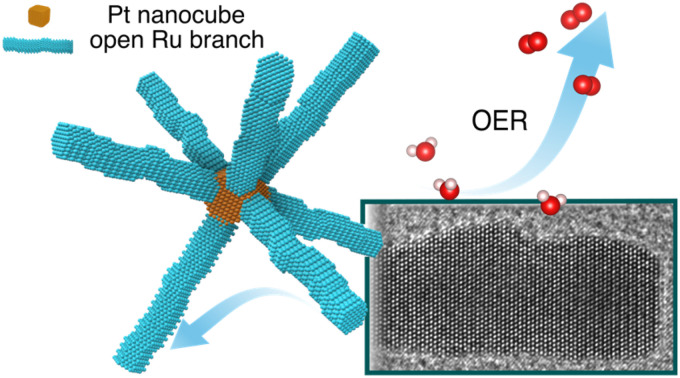

## Introduction

Branched metal nanoparticles are an important class of materials used in sensors,^1^ nanomedicine,^2^ energy conversion,^3^ and optical devices^4,5^ because they combine nanosized dimensions with open structures. This is particularly important for electrocatalysis because (i) nano-dimensions ensure high surface areas are achieved,^6^ (ii) open structure enables these surfaces to be highly accessible to incoming reagents.^7^ Synthesis of metal nanomaterials with open structures is challenging, as highly branched nanoparticles typically form high energy surfaces that can be highly active for electrocatalysis but have limited stability.^8^

Achieving both high activity and stability is a key target for electrocatalysis, particularly in acidic oxygen evolution reaction (OER) where catalysts have long suffered from an activity–stability trade off: Ru is the most highly active metal for OER but requires additional structural modification to prevent the rapid dissolution of catalytically-active Ru^>4+^ species that erode the catalyst.^9,10^ In recent years, the exposure of low-index facets on nanoparticle surfaces has emerged as an effective means of achieving both high activity and stability.^11^ Low-index facets of Ru nanoparticles, such as the (0001) facets, can form the active Ru^>4+^ species but these are highly coordinated to prevent dissolution during catalysis.^12^ One challenge with these systems was the potential for the trapping gas, generated in the electrochemical reaction, being trapped between the slowly spaced branches.^13^ If open structures with low-index facetted Ru branches could be created, the transport of incoming reagents and outgoing O_2_ gas bubbles could be maximised to enhance activity while retaining high stability.

Various open structured nanoparticles, such as fractal,^14^ porous,^15,16^ framework,^17,18^ and branched nanostructures^19,20^ have been successfully synthesised. However, precise control over key factors like architecture, dimensions, and openness remains challenging. In multi-branched structures with more than five branches, limited branch spreading often leads to overlap.^12,20^ Therefore, it is important to develop rational bottom-up design for creating new open structures. A notable example involves controlling the growth orientation by growing hexagonal close-packed (hcp) branches from specific facets of spherical face-centred cubic (fcc) seeds through epitaxial growth.^21^ By further spatially isolating these specific facets on the fcc seeds, we may predict the growth orientation, spatial arrangement, and even the number of branches.

In this work, we present an approach to control the direction and growth of Ru branches using cube-shaped fcc Pt seeds to create highly open and tuneable low-index facetted Ru branched nanoparticles. Pt cube cores were chosen because Pt adopts a fcc structure that enables epitaxial growth with a small difference in lattice spacing of just 3% between the atoms on the Pt {111} with Ru {0001} and because it is relatively easy to grow Pt cubes large enough to enable 8 arms to grow off the central cube. Our previous studies on spheres have shown that seed size can play an important role in controlling the number and diameter of the branches growing off a central seed.^22^ The tunability of the approach is further illustrated by synthesising highly monodisperse nanoparticle samples with precisely controlled branch lengths, tuned between 10 nm to 52 nm. We demonstrate that this approach creates high surface area, highly accessible open, branched Ru nanostructures with low-index facets that are highly active and stable for OER.

## Results and discussion

**Fig. 1:**
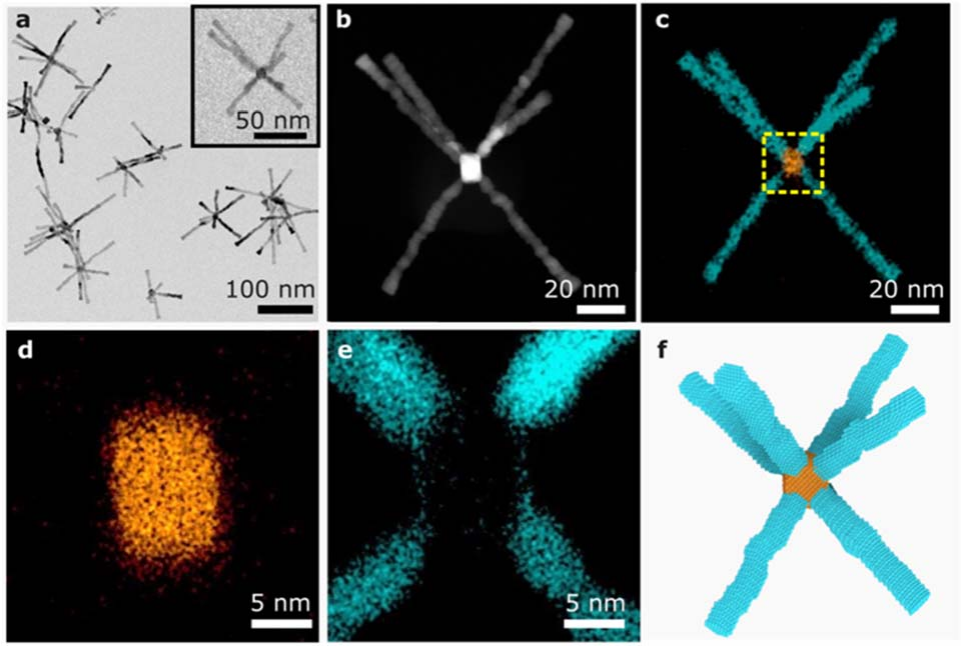
(a) TEM image of open Ru branched nanoparticles. (Inset) An individual nanoparticle for STEM-EDX analysis. (b and c) HAADF-STEM image and EDX mapping of an open Ru branched nanoparticle with Ru branches (cyan) and a Pt cubic core (orange). (d and e) Magnified EDX images of the open Ru branched nanoparticle centre showing Ru branches (cyan) extending from the corners of a Pt nanocube (orange). (f) Model of the open Ru branched nanoparticle.

The open Ru branched nanoparticles were synthesised by the slow growth of Ru onto cube-shaped Pt seeds. Monodispersed Pt nanocubes, averaging 11.6 ± 0.8 nm in size, were used as seeds (Fig. S1†). Slow, controlled growth of Ru onto the seeds was achieved by reacting ruthenium acetylacetonate, dodecylamine and Pt nanocubes in a bottle filled with 2 bars of H_2_ for 72 h. The resulting Ru branches are 52 ± 13 nm in length and arranged in a regular square prismatic geometry around the cores (Fig. 1a and S2a†).

Growth from cubic Pt seeds creates an open branched nanostructure where Ru branches are arranged regularly around a central core. The Ru branches align with the corners of the Pt nanocubes, as shown by high-angle annular dark-field scanning transmission electron microscope (HAADF-STEM) and energy dispersive X-ray spectroscopy (EDX) mapping and spectrum analysis (Fig. 1b, c, S2b, c, and S3a, b†). No Ru shell is observed around the Pt cores (Fig. 1d, e and S2d, e†), indicating Ru branches grow directly off the corners. A model of Ru branches extending from the corners of the Pt cubic core displays branch orientations that match with the morphologies observed in TEM images (Fig. 1f).

**Fig. 2:**
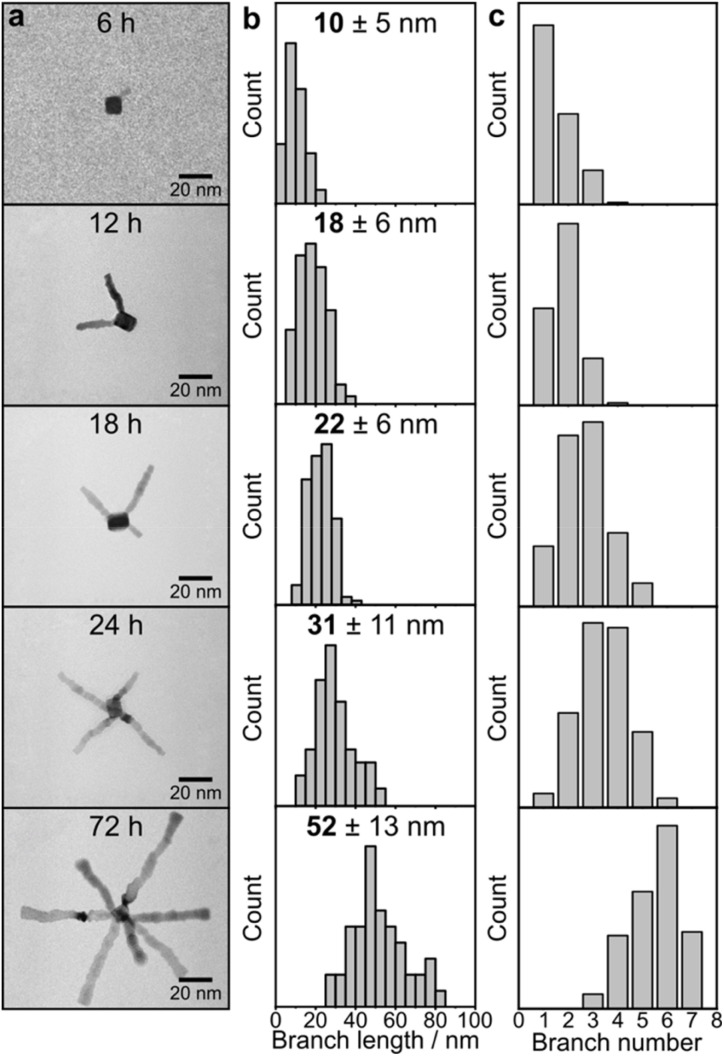
(a) TEM images of open Ru branched nanoparticles with a branch length of 10 nm, 18 nm, 22 nm, 31 nm and 52 nm and their corresponding (b) statistical analysis of branch length and (c) number of branches.

The length of the Ru branches can be controlled by varying the reaction time and amount of Ru precursor. Using the one-fifth of the same Ru precursor, the branch length increased from 10 ± 5 nm to 18 ± 6 nm, 22 ± 6 nm, and 31 ± 11 nm with different reaction times of 6 h, 12 h, 18 h, and 24 h, respectively (Fig. 2a and b). Interestingly, the number of branches also increases with longer reaction times, with 2 branches per nanoparticle observed at 6–12 h, followed by 3 branches at 18–24 h and 7 branches at 72 h (Fig. 2c). The branch width remains constant around 4 nm to 5 nm from 6 h to 72 h, indicating that there is no growth occurring on the sides of branches as the reaction continues (Fig. S4†).

**Fig. 3:**
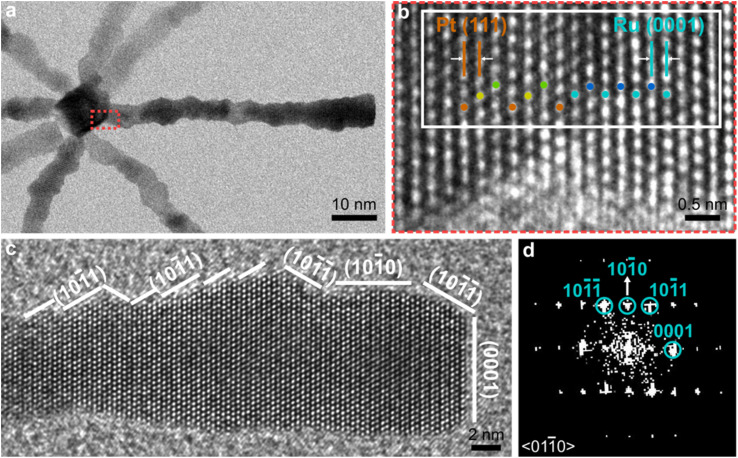
(a) TEM image of the core area of an open Ru branched nanoparticle. (b) HRTEM of the joint of Pt core and Ru branch (red box in (a)). The interface between the Pt corner and Ru branch shows ABCABC stacking in the Pt (111) plane and ABABAB stacking in Ru (0001) plane. Orange, yellow, and green spots represent atoms with A, B, and C stacking in fcc Pt nanocubes, respectively. Cyan and blue spots represent atoms with A and B stackings in hcp Ru branches, respectively. (c) HRTEM image of a Ru branch, showing the expose of Ru low-index facets on the surface. (d) FFT taken from the Ru branch in (c). The spots match an hcp-structured Ru viewed down the 〈011̄0〉 zone axis.

Selected area electron diffraction (SAED) confirms the presence of fcc Pt and hcp Ru (Fig. S5†). High resolution transmission electron microscope (HRTEM) imaging of the core-branch interface further shows that the hcp Ru (0001) branch lattice planes are epitaxially aligned with the fcc Pt (111) core lattice planes (Fig. 3a and b). The transition from the ABCABC stacking sequence in fcc Pt (111) to an ABABAB sequence in hcp Ru (0001) occurs without stacking faults (Fig. 3b and S6a†). Direct growth of Ru branches off the corners of the Pt nanocubes is possible due to the low lattice mismatch of 3% between Pt (111) 2.27 Å and hcp Ru (0001) 2.34 Å. This low mismatch minimises strain at the core-branch interface, as shown by the alignment of hcp (0001) and fcc (111) reflections and the absence of streaking in the Fast Fourier transforms (FFTs) images of the core-branch interface (Fig. S6b and c†).

Use of densely packed long chain amine, dodecylamine, as a surfactant enables the formation of low-index facets on the surfaces.^23^ The surface facets were indexed as {101̄0} and {101̄1} facets on the branch side and (0001) on the branch tips (Fig. 3c, d and S7a, b†). The reversible binding of dodecylamine to the branch surface enables Ru atoms to add to energised sites, resulting in low energy facets with high coordination numbers.^24–26^ These low-index facets and defect-free connection between the Pt core and Ru branches, are both highly stable and minimise the number of potential sites where active Ru species can be dissolved during catalysis.^27^

**Fig. 4:**
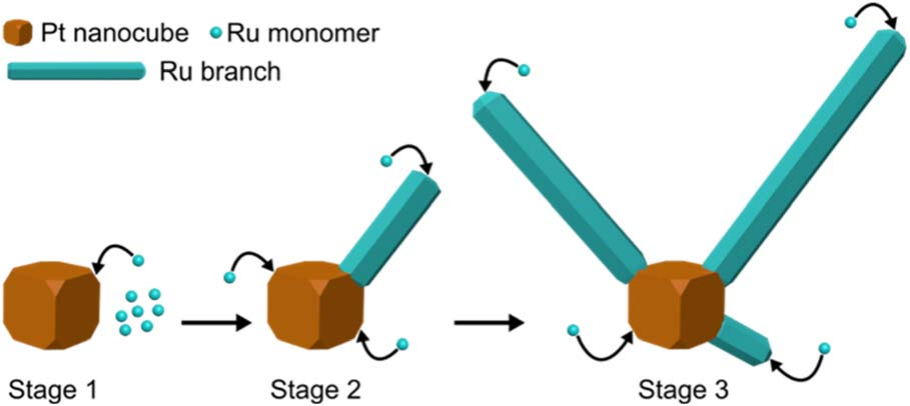
Growth mechanism of the open Ru branched nanoparticles: stage (1) nucleation of Ru at the corner of Pt nanocubes, stage (2) nucleation of Ru at the tip of a Ru branch and other corners of Pt nanocubes, and stage (3) increase of Ru branch numbers and lengths, forming an open structure.

The growth mechanism of Ru branches on the cubic Pt seeds occurs in three stages, as illustrated in Fig. 4. In the first stage, Ru precursor is reduced, and form Ru atoms in solution. The nucleation of Ru occurs exclusively at the highly exposed and energetic corners of Pt nanocubes. In the second stage, incoming Ru atoms can add to either of the two high energy and exposure sites, that is (i) the tips of branches, resulting in branches growing longer or (ii) the corners on the Pt nanocubes, resulting in the nucleation of additional branches. The increase in both branch length and number as reaction time is increased suggests that both growth processes are occurring simultaneously. In the final stage, the Ru branches keep growing longer and branch numbers increase.

Despite nucleation events occurring at different times, the nanoparticles formed at all reaction times have a narrow distribution of branch lengths. This indicates that branches that form from later nucleation events grow more rapidly than branches formed earlier in the reaction.^28,29^ This observation aligns with the tendency for multilayer Ru islands to form on Pt(111), as the Ru–Ru bond is stronger than the Ru–Pt bond.^30^

The preformed Pt cubic cores are essential for forming the open structure. In the absence of Pt seeds, pure Ru branches averaging 28 nm in length and 4.4 nm in width form (Fig. S8†).^12^ These compact and overlapping structures may trap O_2_ gas generated during the electrocatalysis between the branches.^13^ This comparison with open Ru branched nanoparticles provides insights into the relationship between electrocatalytic activity and branched structure.

The impact of branch length and open branched structure on OER performance was assessed by comparing the electrocatalytic properties of open Ru branched nanoparticles with 52 nm branches (Fig. S9b†) and 31 nm branches (Fig. S9c†) to pure Ru nanoparticles with overlapping branches (Fig. S9d†). The electrocatalytic OER activity were evaluated using a three-electrode system with rotating disk electrode in O_2_-saturated 0.1 M HClO_4_ electrolyte, with carbon supported nanoparticles as the working electrode.

**Fig. 5:**
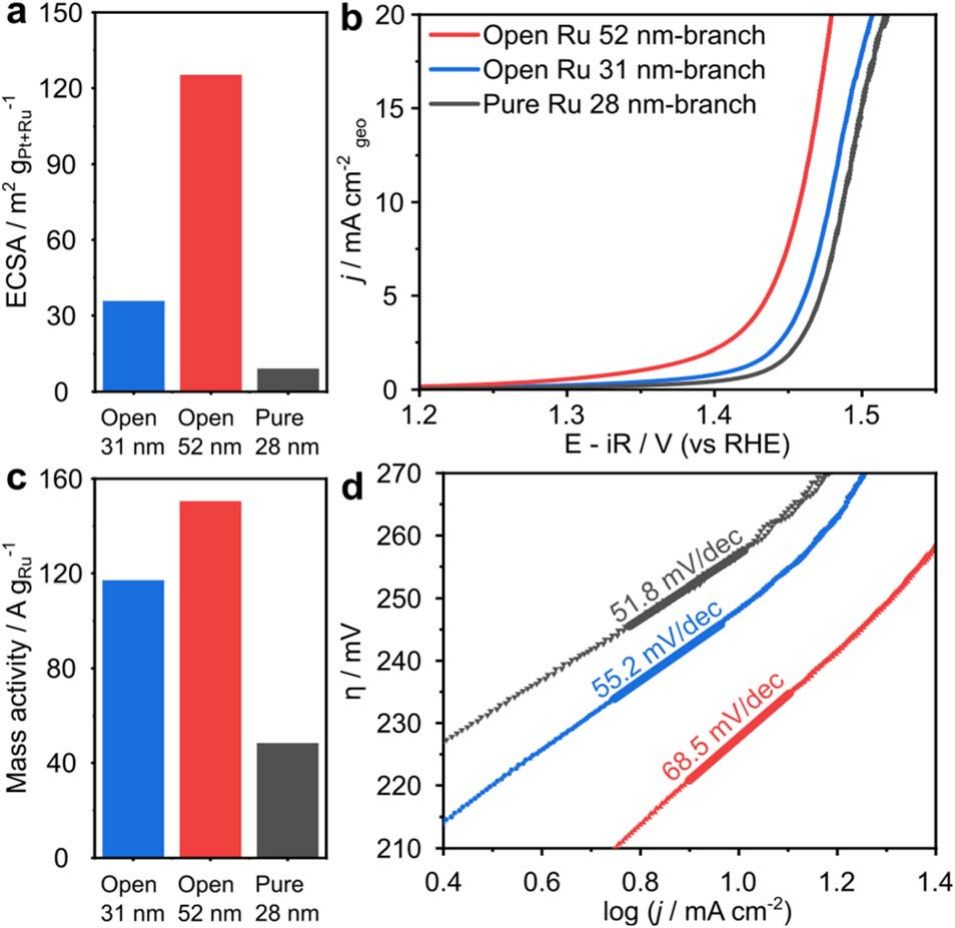
(a) ECSA comparison of the branched Ru nanoparticles. (b) LSV curves of the open Ru 52 nm-branch (red line), open Ru 31 nm-branch (blue line), pure Ru 28 nm-branch (grey line) in an O_2_-saturated 0.1 M HClO_4_. (c) Mass activities comparison of the branched Ru nanoparticles at 1.48 V *vs.* RHE. (d) Tafel slope comparison of the branched Ru nanoparticles derived from (b).

The open Ru 52 nm-branched nanoparticles have electrochemically active surface areas (ECSAs) of 125.3 m^2^ g^−1^ that are 3.5× and 14× greater than the open Ru 31 nm-branched nanoparticles and pure Ru nanoparticles, respectively (Fig. 5a, S10 and Table S3†). The pure Ru-28 nm-branch nanoparticles exhibit a significantly lower ECSA of 9.2 m^2^ g^−1^ due to their compact and overlapping branched structure. This validates that open structures enable greater exposure and accessibility to surface and result in higher ECSA.^7,31^ The open structures create catalysts with very high ECSAs compare with other state-of-the-art Ru OER catalysts (Table S1†).

The open Ru 52 nm-branched nanoparticles reach a geometric current density of 10 mA cm^−2^ at a lowest overpotential of 227 mV (Fig. 5b and S11a, c†). This is 80 mV lower than state-of-art RuO_2_ catalysts, and competitive with other reported Ru catalysts (Table S1†).^32,33^ The negligible OER activity of Pt nanocubes indicates that open Ru branches are the only active sites (Fig. S11c and d†).^34^

The mass activities of the nanoparticles assessed in this study follow the same trend as ECSA (Fig. 5c); the open Ru 52 nm-branched nanoparticles achieve a mass activity of 150.5 mA mg^−1^ at 1.48 V, which is 1.3× higher than the open Ru 31 nm-branched nanoparticles (117.1 mA g^−1^) and 3.1× higher than the pure Ru nanoparticles (48.4 mA g^−1^). This indicates that an open structure enhances the catalytic performance by exposing more electrochemically active Ru sites and facilitating mass transport during the OER process.

Additionally, the Tafel slope of open Ru 52 nm-branch is 68.5 mV dec^−1^ (Fig. 5d), which is lower than that of commercial RuO_2_ (83.8 mV dec^−1^, Fig. S11b†).^35^ The Tafel slope around 60 mV dec^−1^ indicates that the rate-determining step involves the rearrangement of bonded OH species *via* a surface reaction.^36^

Such an open branched structure exposing low-index facets also exhibits superior stability evaluated by the method of chronopotentiometry at a constant current density of 5 mA cm^−2^. The open Ru 52 nm-branch shows the stability for 300 min with a decay around 400 mV, outperforming pure Ru 28 nm-branch and open Ru 31 nm-branch (Fig. S12†). The significant difference in stability could also be attributed to more active sites and more efficient mass transfer, reducing the burden on each active sites. The stability of the open Ru 52 nm-branch is comparable to that of other state-of-art Ru-based OER catalysts (Table S1†). The loss of activity of the open Ru 52 nm-branch nanoparticles was further examined by post-catalysis TEM after chronopotentiometry testing for 300 min. The sample contains a mixture of nanoparticles, including relatively intact structures (Fig. S13a†) and nanoparticles with partially dissolved Ru branches and an intact cubic Pt core (Fig. S13b†). However, most of the sample were Pt nanocubes with the Ru branches fully dissolved (Fig. S13c†). These observations show that the loss of activity is due to the dissolution of active Ru branches.

## Conclusions

In conclusion, we demonstrate that directionally controlled growth of branches from well-defined shaped seeds is an effective means of synthesising open Ru branched nanostructures with low-index facets. The tunability of the approach is illustrated by synthesising highly monodisperse nanoparticle samples with precisely controlled branch lengths, tuned between 10 nm to 52 nm. The open branched structure is key to overcoming the activity–stability trade-off, as it enables the stable facetted surfaces to be highly accessible to incoming reagents and achieves high electrochemically active surface areas. This concept unlocks a new opportunity to synthesise a range of branched nanoparticles with a desired open structural feature useful for many catalytic applications. Future studies will include adapting this approach to other fcc structured cubes such as nickel and copper and hcp structured branches such as cobalt.

## Data availability

The data that support the findings of this study are available in the ESI† of this article and are available on request from the corresponding authors.

## Author contributions

S. X.: data curation, formal analysis, investigation, writing – original draft, writing – review & editing; Y. X.: conceptualization, methodology, writing – review & editing; A. R. P.: conceptualization, writing – review & editing; L. G.: conceptualization, methodology, writing – review & editing; Q. L.: methodology, writing – review & editing; S. C.: methodology; Z. R. R., I. P., Y. S.: methodology, writing – review & editing; D. L. H.: writing – review & editing; L. D.: funding acquisition, writing – review & editing; J. J. G.: conceptualization, funding acquisition, resources, supervision; R. D. T.: conceptualization, funding acquisition, project administration, resources, supervision.

## Conflicts of interest

The authors declare no competing financial interest.

## Supplementary Material

SC-OLF-D5SC01861G-s001
