# Supplementary material for: Formation of open ruthenium branched structures with highly exposed active sites for oxygen evolution reaction electrocatalysis
Source: Chem Sci. 2025 Apr 16;16(21):9284–9. doi: 10.1039/d5sc01861g (PMC12022671; doi:10.1039/d5sc01861g)
Supplement: SC-016-D5SC01861G-s001 [file SC-016-D5SC01861G-s001.pdf]

Supplementary Information (SI) for Chemical Science.
This journal is © The Royal Society of Chemistry 2025

Supporting Information

Formation of Open Ruthenium Branched Structures with Highly Exposed Active Sites for Oxygen Evolution Reaction Electrocatalysis†

Sa Xiao,[a] Yuhan Xie,[a] Agus R. Poerwoprajitno,[b] Lucy Gloag,[c] Qinyu Li,[a] Soshan Cheong,[d] Zeno R. Ramadhan,[a] Ingemar Persson,[a] Yoshiki Soda,[a] Dale L. Huber,[b] Liming Dai,[e] J. Justin Gooding *[af] and Richard D. Tilley *[adf]

[a.] *School of Chemistry, The University of New South Wales, Sydney, NSW 2052, Australia.*

[b.] *Center for Integrated Nanotechnologies, Sandia National Laboratories, Albuquerque, NM 87185, USA.*

[c.] *Research School of Chemistry, The Australian National University, Canberra, ACT 2601, Australia.*

[d.] *Mark Wainwright Analytical Centre, The University of New South Wales, Sydney, NSW, 2052, Australia.*

[e.] *School of Chemical Engineering, The University of New South Wales, Sydney, NSW, 2052, Australia.*

[f.] *Australian Centre for NanoMedicine, The University of New South Wales, Sydney, NSW, 2052, Australia.*

* Corresponding author: justin.gooding@unsw.edu.au, r.tilley@unsw.edu.au

Materials. Platinum (II) acetylacetonate ($Pt(acac)_2$, 98%), oleylamine (technical grade, 70%), oleic acid (90%), tungsten hexacarbonyl ($W(CO)_6$, 97%), ruthenium acetylacetonate ($Ru(acac)_3$, 97%), dodecylamine (DDA, 98%), mesitylene (98 %) and 5% Nafion 117 containing solution were purchased from Sigma-Aldrich. Toluene, ethanol (96%), hexane, and isopropyl alcohol (99.5%) were purchased from Chem-Supply Pty. Ltd. Perchloric acid (70%) was purchased from Suprapur-Merck. Vulcan carbon black (XC-72R) and commercial RuO_2 catalyst were purchased from Fuel Cell Store. Milli-Q water (DI water, resistivity >18.3 MΩ•cm) was used to prepare electrolytes.

Synthesis of Pt nanocubes. Pt nanocubes were synthesized using a previously reported procedure.[1] $Pt(acac)_2$ was dispersed in a solution of oleylamine (4 mL) and oleic acid (1 mL) in a 25 mL two-neck flask connected with a condenser. The mixture was then stirred gently and heated to 130 °C within 30 min under an Ar environment. $W(CO)_6$ (25 mg) was added into the solution after the temperature reached 130 °C. The solution was then heated to 240 °C within 15 min and kept at 240 °C for 40 min. Finally, the reaction was cooled down to room temperature and the particles were washed twice using a 1:2 mixture of ethanol and n-hexane centrifugation at 3500 rpm for 5 min.

Synthesis of open branched Ru nanoparticles. The open branched Ru nanoparticles were synthesized by modifying a previously published procedure from our group.[2] For the synthesis of open Ru 31 nm-branch nanoparticles, platinum nanocubes (0.005 mmol), $Ru(acac)_3$ (0.01 mmol), and DDA (0.5 mmol) were dispersed in mesitylene (4 mL). The solution was then transferred to a Fischer-Porter bottle, filled with 2 bars of H_2 gas before sealing and placing in an oil bath at 140 °C. After 24 h, the bottle was removed and cooled down to ambient temperature before releasing the residential gas. The black solution was transferred to centrifuge tube, washed twice using a 1:1 mixture of toluene and ethanol at 3000 rpm for 5 min. The purified nanoparticles were redispersed in toluene. To investigate the growth mechanism, time-resolved experiments were carried out by repeating experiments and quenching the reaction at 6 h, 12 h, and 18 h respectively. The synthesis of open Ru 52 nm-branch nanoparticles was achieved using the same protocol, but the amount of $Ru(acac)_3$ was increased from 0.01 mmol to 0.05 mmol and reaction time increased to 72 h.

Synthesis of pure branched Ru nanoparticles. The pure Ru 28 nm-branch nanoparticles were synthesized by modifying a previously published procedure from our group.[3] $Ru(acac)_3$ (0.1 mmol), DDA (1 mmol) were dissolved in 1-octadecene (2.0 mL). The solution was then transferred to a Fischer-Porter bottle, filled with 3 bars of H_2 gas before sealing and placing in an oil bath at 145 °C. After 48 h, the bottle was removed and cooled down to ambient temperature before releasing the residential gas. The resulting nanoparticles was transferred to

centrifuge tube, washed twice using a 1:1 mixture of toluene and ethanol at 3000 rpm for 5 min. The purified nanoparticles were redispersed in toluene.

Characterizations. Samples were prepared for transmission electron microscopy (TEM) characterization by drop casting the nanoparticle suspension in toluene onto carbon-coated copper grids and air-drying it. Low-resolution TEM, high-resolution TEM (HRTEM), scanning TEM (STEM), selected area electron diffraction (SAED), and energy dispersive X-ray (EDX) mapping were performed on a JEOL JEMF200 FEG transmission electron microscope operated at 200 kV equipped with an annular dark field (ADF) detector and a JEOL windowless 100 mm^2 silicon drift X-ray detector. The Ru : Pt ratios of the as-prepared open branched Ru nanoparticles were determined by EDX spectroscopy, which are summarized in Table S2. The length of the Ru branches was analyzed on 100 nanoparticles by measuring the distance between the tip of the Ru branch to the interface between the Ru branch and the Pt nanocubes.

Electrochemical measurement. The electrochemical tests were performed on a CHI-660E Potentiostat with a three-electrode cell setup. An aqueous solution of 0.1 M $HClO_4$ prepared with deionized water was used as the electrolyte. An Ag/AgCl (3 M KCl) was used as a reference electrode and a platinum plate as the counter electrode. All potentials reported in this work were referenced to the reversible hydrogen electrode (RHE) scale with 95% iR compensation with the following equation, where 0.201 V is the potential of reference electrode (Ag/AgCl) measured against standard hydrogen electrode (1 M HCl), i is the measured current and R is the uncompensated resistance determined by electrochemical impedance spectroscopy. E (vs. RHE) = E (vs. Ag/AgCl) + 0.201 V + 0.059 V × pH – 95% iR.

The as-synthesized Pt nanocubes and branched Ru nanoparticles were loaded onto carbon black (Vulcan XC-72R) with a metal loading of 20 wt.% by dispersing surfactant stabilized nanoparticles and carbon black in hexane and sonicating for 2 h. After drying, the carbon loaded nanoparticles were placed in a tube furnace at 300 °C for 6 h in argon flow to remove organic surfactants.[4] The catalyst ink was prepared by mixing 1 mg of the carbon supported nanoparticles in 200 μL solution of 70 vol % deionized water, 29 vol % isopropanol, and 1 vol % Nafion solution (5 wt %). The mixture was sonicated for 30 min to obtain a homogeneous ink. The catalyst ink for commercial RuO_2 was prepared using the same recipe. An aliquot of 30 μL was dropped onto a 5-mm-diameter glassy-carbon rotating disk electrode (0.196 cm^2, Pine Research, Model AFE5TQ050PK). The metal catalysts loading was calculated to be 0.153 mg/cm^2. The corresponding Pt and Ru masses of the as-prepared nanoparticles used in OER catalysis are summarized in Table S2. The electrode was then air dried before electrochemical measurements. The OER measurements was performed in O_2 saturated 0.1 M $HClO_4$ at a sweep rate of 50 mV s^{-1}

while rotating the working electrode at 1600 rpm. The mass activity of the branched Ru catalysts was determined using the equation of $j_{mass\ activity} = i$ / m, where i is the current, and m is the Ru mass loading in open Ru 31 nm-branch, 52 nm-branch, and pure 28 nm-branch respectively. For the open Ru 31 nm-branch and 52 nm-branch nanoparticles, the Ru mass is determined based on the Ru fraction in the total mass (Pt + Ru), as derived from the EDX spectrum. The electrochemical active surface area (ECSA) was obtained by CO stripping by applying 0.1 V (vs. RHE) for 5 minutes in CO-saturated 0.1 M $HClO_4$ followed by 3 cycles of cyclic voltammetry at 10 mV s^{-1}. The ECSA was then deduced from CO oxidation peak using the equation of ECSA = Q_{co} / q_{co}, where Q_{co} is the charge for CO oxidation and q_{co} (420 μC cm^{-2}) is the conversion factor. The ECSA of the as-prepared nanoparticles used in OER catalysis are summarized in Table S3. The stability test was conducted using the chronopotentiometry method at a constant geometric current density of 5 mA cm^{-2}. The working electrode was prepared by coating carbon carbon cloth (1 × 1 cm^2) with 150 μL of catalyst ink, which gives the same catalysts loading of 0.153 mg/cm^2 as the glassy-carbon rotating disk electrode.

Table of Contents

Figure S1 | TEM image of Pt nanocubes used as seeds.

Figure S2 | TEM image, HAADF-STEM, and EDX maps of open Ru 31 nm-branch nanoparticles.

Figure S3 | EDX spectra of the open Ru 31 nm-branch and open Ru 52 nm-branch nanoparticles.

Figure S4 | TEM images of open branched Ru nanoparticles with a branch length of 10 nm, 18 nm, 22 nm, 31 nm and 52 nm, and their corresponding branches width and numbers.

Figure S5 | SAED of open 52 nm-branch nanoparticles and indexing results.

Figure S6 | HRTEM of the joint of Pt core and Ru branch, and their respective FFT images.

Figure S7 | HRTEM and FFT image of a single branch of open Ru 31 nm-branch nanoparticles.

Figure S8 | TEM images of pure Ru 28 nm-branch nanoparticles and statistical analysis of branch length and width.

Figure S9 | TEM image of carbon supported Pt nanocubes, and open Ru 31 nm-branch, open Ru 52 nm-branch and pure 28 nm-branch nanoparticles.

Figure S10 | Electrochemical active surface area (ECSA) measurement of open Ru 31 nm-branch, open Ru 52 nm-branch, pure Ru 28 nm-branch nanoparticles and Pt nanocubes.

Figure S11 | LSV curve and Tafel plot of the Pt nanocube and commercial RuO_2.

Figure S12 | Chronopotentiometry performance of branched Ru nanoparticles.

Figure S13 | TEM images of the open Ru 52 nm-branch sample after the chronopotentiometry test.

Table S1 | Summary of recently reported Ru-based catalysts for acidic OER using a three-electrode system with rotating disk electrode.

Table S2 | The Pt and Ru ratios and masses of the as-prepared nanoparticles used in OER catalysis.

Table S3 | Comparison of calculated electrochemical surface areas (ECSAs) obtained from CO stripping for Pt nanocubes and branched Ru nanoparticles.

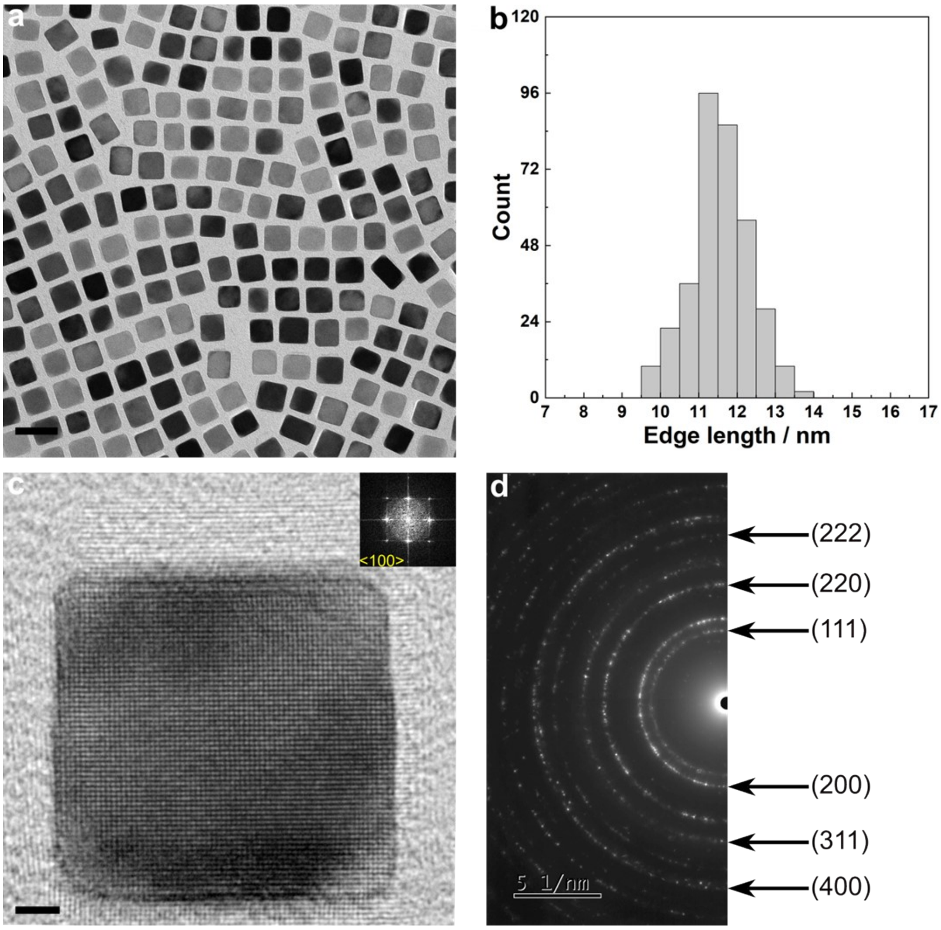

Figure S1. a) TEM image of Pt nanocubes. Scale bar = 20 nm. b) Size distribution of Pt nanocubes, with the average edge length of 11.6 ± 0.8 nm. c) HRTEM image of a Pt nanocube. Scale bar = 2 nm. d) Electron diffraction pattern of Pt nanocubes.

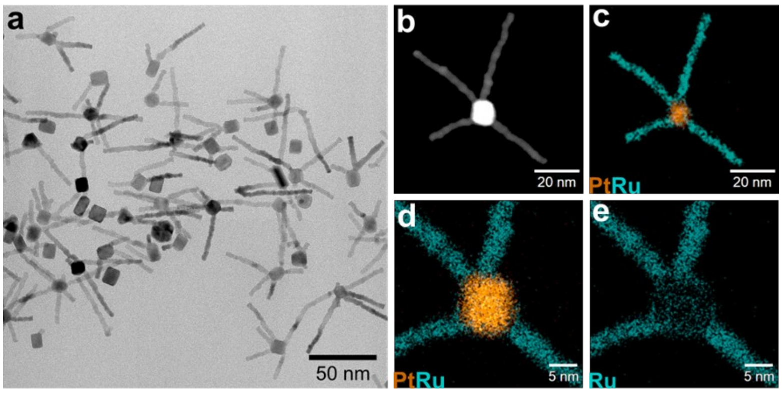

Figure S2. a) TEM image of open Ru 31 nm-branch nanoparticles. b-e) HAADF-STEM image and EDX maps of an open Ru 31 nm-branch nanoparticle with Ru branches and partial shell grown off the Pt nanocubes.

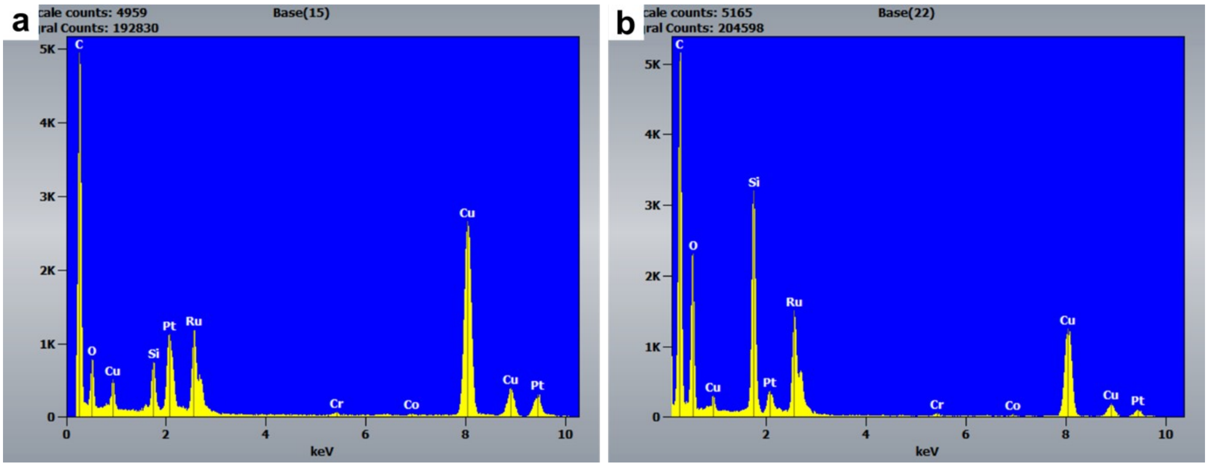

Figure S3. EDX spectrum of the a) open Ru 31 nm-branch nanoparticles and b) open Ru 52 nm-branch nanoparticles. The Ru : Pt ratios, as determined by EDX spectroscopy, are 1.5 : 1 and 7.3 : 1 for the open Ru 31 nm-branch and open Ru 52 nm-branch, respectively.

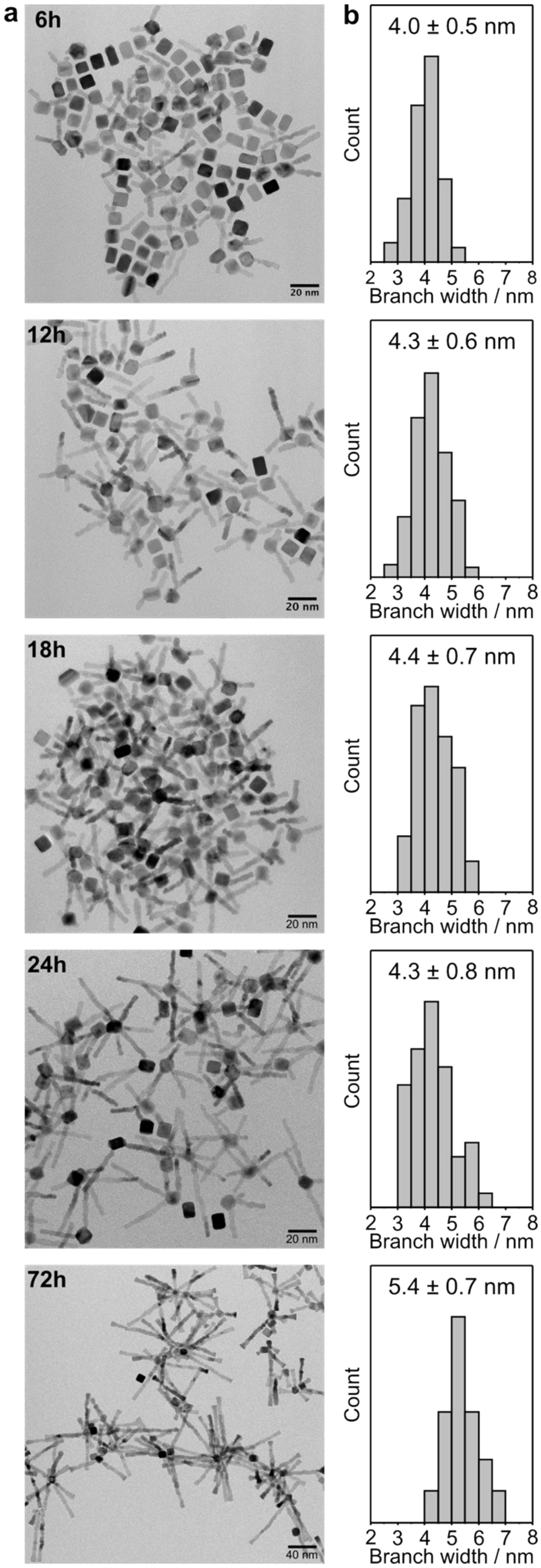

Figure S4. a) TEM images of open branched Ru nanoparticles with a branch length of 10 nm, 18 nm, 22 nm, 31 nm and 52 nm synthesized at different reaction time and their corresponding, b) branch width.

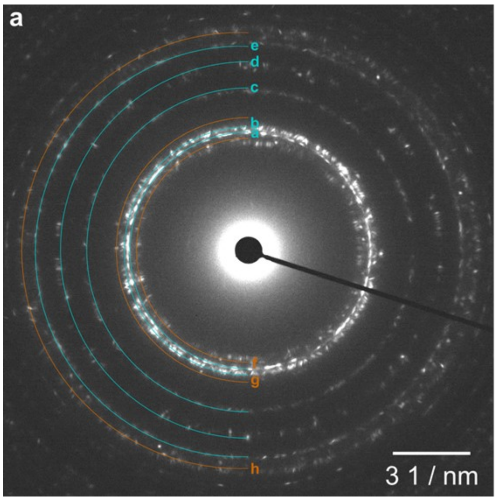

b

Electron diffraction rings	Measured d (Å)	*hcp* Ru (Å)	hkl	*fcc* Pt (Å)	hkl
a	2.144	2.134	0002		
b	2.048	2.055	$10\bar{1}1$		
c	1.564	1.578	$10\bar{1}2$		
d	1.366	1.354	$2\bar{1}\bar{1}0$		
e	1.23	1.216	$10\bar{1}3$		
f	2.266			2.277	111
g	1.956			1.972	200
h	1.126			1.189	311

Figure S5. a) SAED of open 52 nm-branch nanoparticles. b) Indexing shows that the SAED pattern is characteristic of *fcc* Pt and *hcp* Ru.

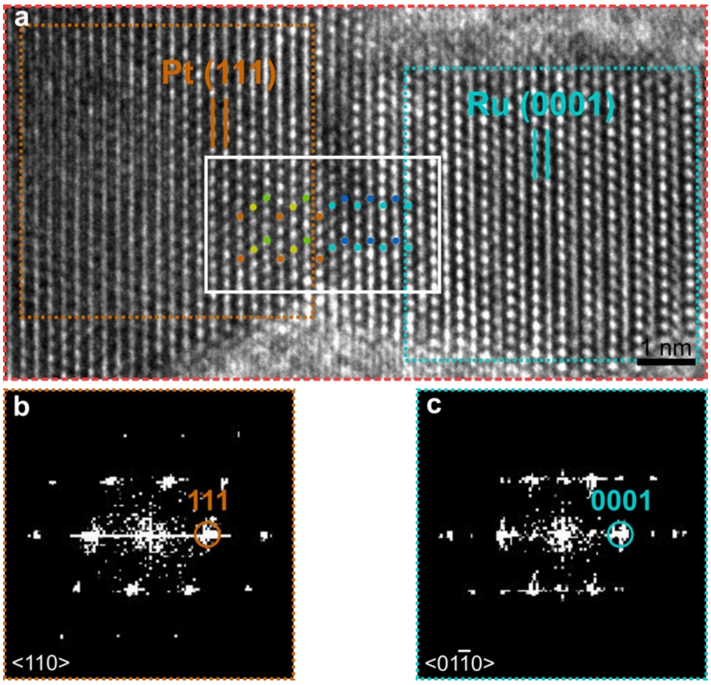

Figure S6. a) HRTEM of the joint of Pt core and Ru branch. The Pt-vertex Ru-branch interface with ABCABC and ABABAB stacking in the Pt (111) and Ru (0001) branch are labelled respectively. Orange, yellow, and green spots indicate atoms with A, B, and C stackings in *fcc*-Pt nanocubes, respectively. Cyan and blue spots indicate atoms with A, B stackings in *hcp*-Ru branches. b) Fast Fourier transform (FFT) of the Pt core (yellow box in a)). The spots match a *fcc* Pt (111) plane from the Pt cubic core viewed down a <110> zone axis. c) FFT of the Ru branch (cyan box in a)). The spots match a *hcp* Ru (0001) plane in the c-axis from the Ru branch viewed down a <01$\bar{1}$0> zone axis.

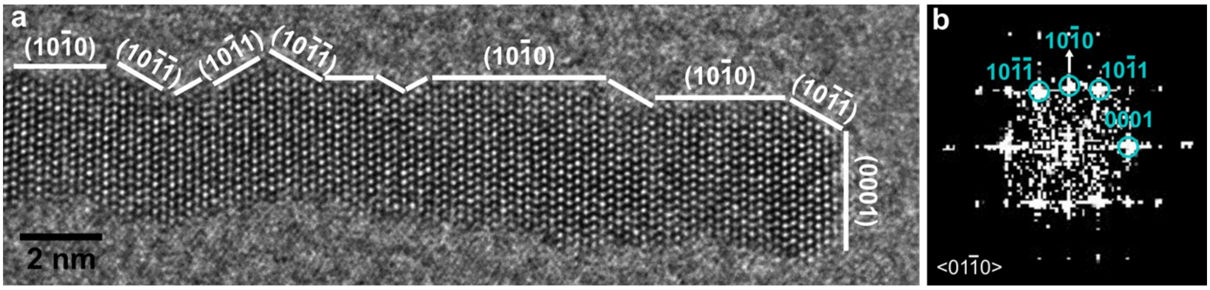

Figure S7. a) HRTEM image of a single branch of an open Ru 31 nm-branch nanoparticle showing the presence of low-index facets. The Ru atoms are arranged in an *hcp* crystal structure viewed down the <01$\bar{1}$0> zone axis. b) FFT of the HRTEM image in a).

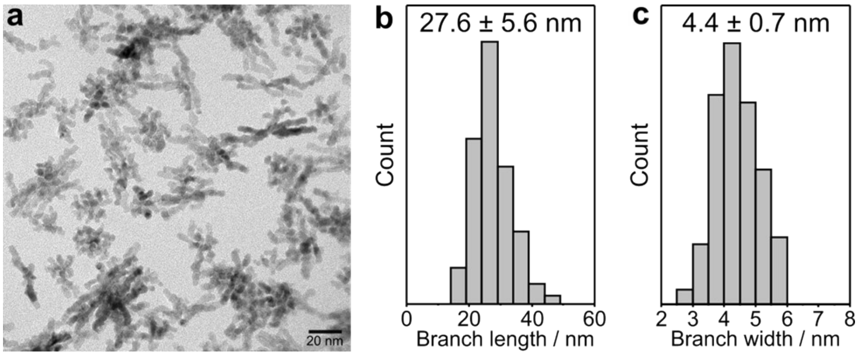

Figure S8. a) TEM images of pure Ru 28 nm-branch nanoparticles synthesized without Pt nanocube core, and their corresponding b) branch length statistical analysis, and c) branch width statistical analysis**.**

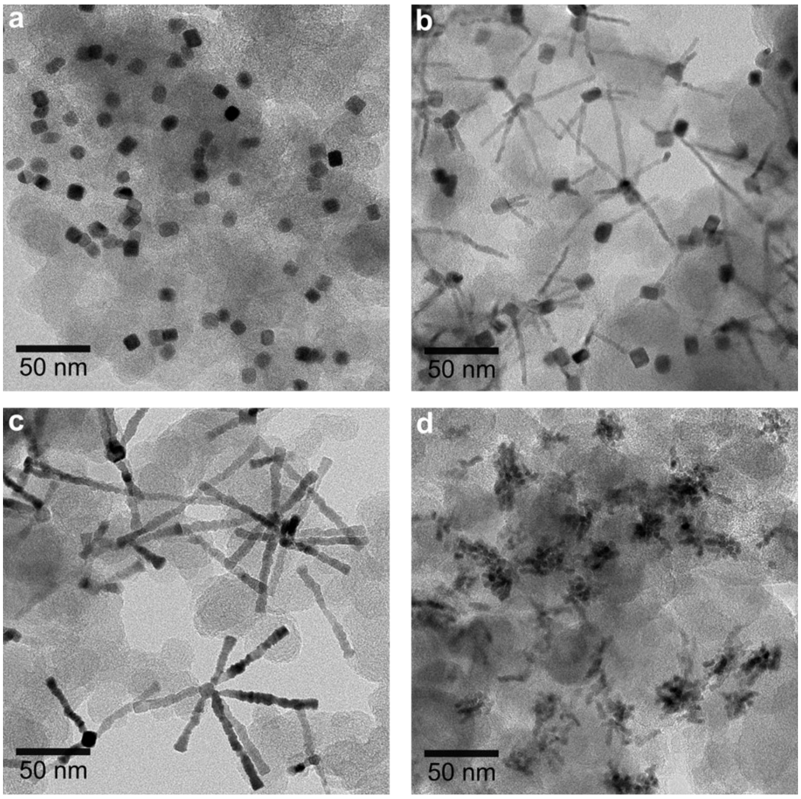

Figure S9. TEM image of carbon supported a) Pt nanocubes, b) open Ru 31 nm-branch nanoparticles, c) open Ru 52 nm-branch nanoparticles, d) pure Ru 28 nm-branch nanoparticles.

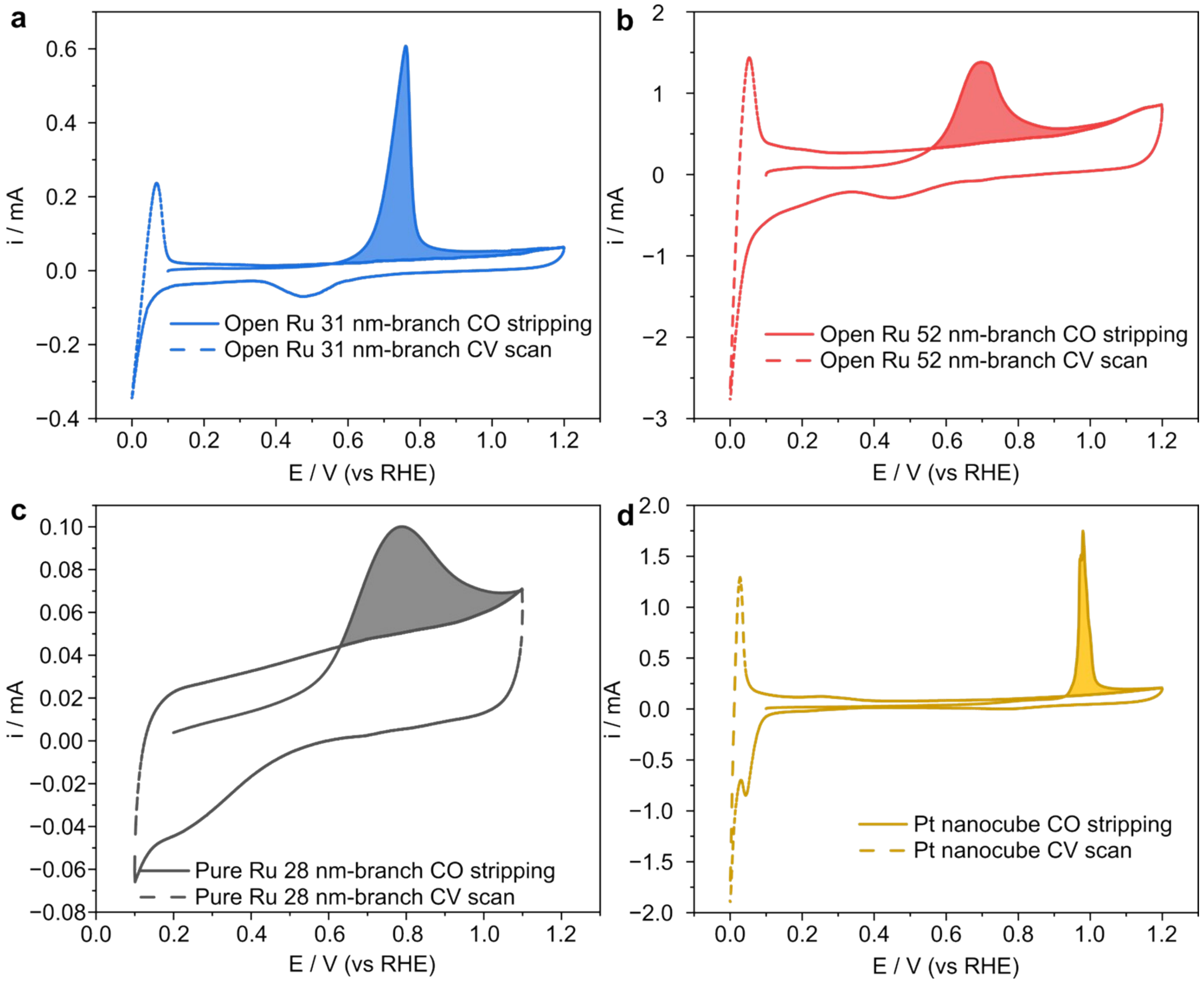

Figure S10. ECSA measurements open Ru 31 nm-branch, open Ru 52 nm-branch, and pure Ru 28 nm-branch using CO-stripping method in 0.1 M CO-saturated $HClO_4$ with a scan rate of 10 mV s^{-1}. a) open Ru 31 nm-branch, b) open Ru 52 nm-branch, c) pure Ru 28 nm-branch, and d) Pt nanocubes.

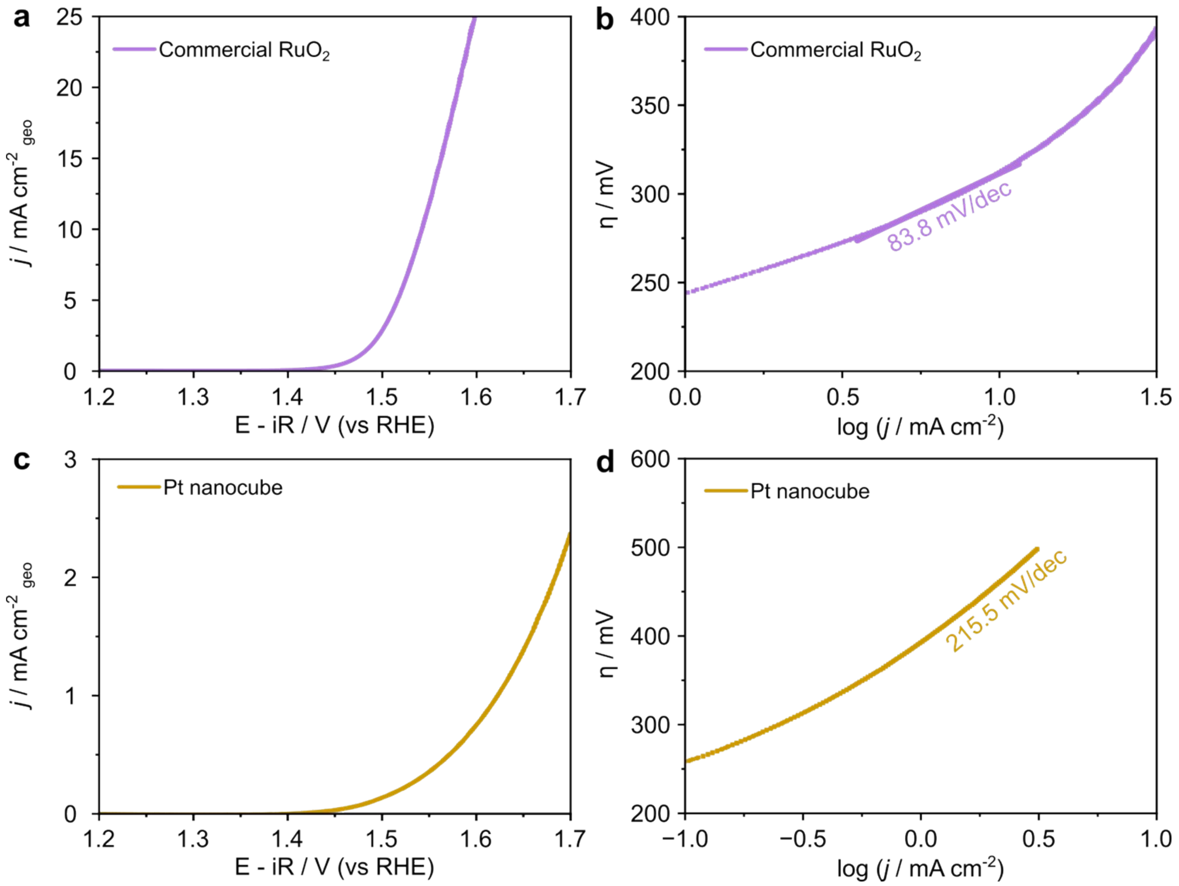

Figure S11. a) LSV curves of commercial RuO_2 in an O_2-saturated 0.1 M $HClO_4$, b) Tafel plots of the commercial RuO_2 derived from a). c) LSV curves of Pt nanocube in an O_2-saturated 0.1 M $HClO_4$, d) Tafel plots of Pt nanocubes derived from c).

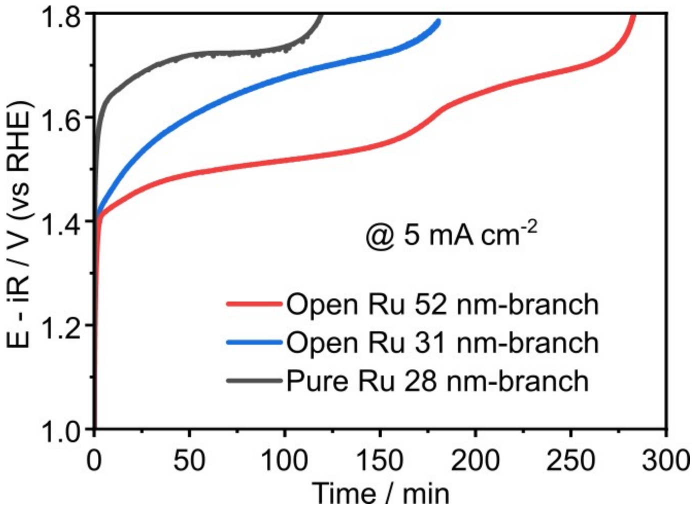

Figure S12. Chronopotentiometry performance of branched Ru nanoparticles under constant geometric current density of 5 mA cm^{-2}.

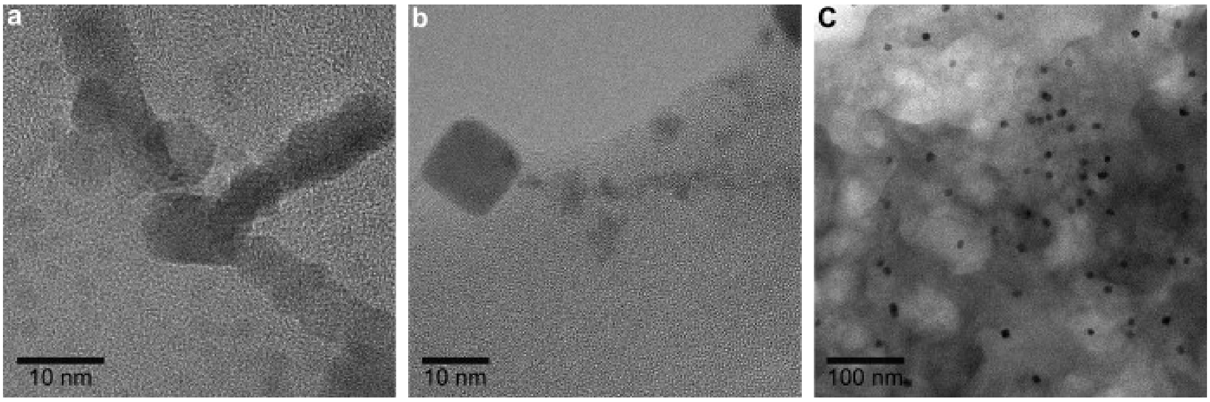

Figure S13. TEM images of the open Ru 52 nm-branch sample after the chronopotentiometry test, a) a nanoparticle with relatively intact structures, b) a nanoparticle with partially dissolved Ru branches and intact cubic Pt core, c) most of the sample are Pt nanocubes without Ru branches.

Table S1. Summary of recently reported Ru-based catalysts for acidic OER using a three-electrode system with rotating disk electrode.

Catalyst	Electrolyte	η @ 10mA cm^{-2} (mV vs RHE)	ECSA (m^2 g^{-1})	Mass activity @ η (A g^{-1} @ mV)	Tafel slope (mV dec^{-1})	Stability	Reference
Open Ru 52 nm-branch	0.1 M $HClO_4$	227	125.3	150.5 @ 1.48	68.5	295 mV @ 2h @ 5 mA cm^{-2}	This work
Open Ru 31 nm-branch	0.1 M $HClO_4$	248	35.8	117.1 @ 1.48	55.2	467 mV @ 2h @ 5 mA cm^{-2}	This work
Pure Ru 28 nm-branch	0.1 M $HClO_4$	256	9.2	48.4 @ 1.48	51.8	493 mV @ 1h @ 5 mA cm^{-2}	This work
Ru_1-Pt_3Cu	0.1 M $HClO_4$	220	75.8	779 @ 1.51	~51.7	~225 mV @ 28 h	[5]
GB-RuO_2	0.1 M $HClO_4$	187	300.6	114.9 @ 1.45	34.5	233 mV @ 140 h @ 10 mA cm^{-2}	[6]
3D Woodpile-structured Ir catalysts	0.05 M H_2SO_4	270	43.6	3760 @ 1.55	42.41	80% activity @ 500 cycles	[7]
Pd@Ir3L	0.1 M $HClO_4$	245	~146.07	3.3 @ 1.63	60.4	276 mV @ 2000 LSVs	[8]
Co-RuIr	0.1 M $HClO_4$	235	-	~19.6 @ 1.465	66.9	235 mV @ 25h @ 10 mA cm^{-2}	[9]
Au-Ru	0.1 M $HClO_4$	220	-	~24.5 @ 1.45	62	33% activity @ 1000 LSVs	[10]
Faceted Ru	0.1 M $HClO_4$	180	-	~86.9 @ 1.41	52	255 mV @ 2h @ 10 mA cm^{-2}	[3]
RuB_2	0.5 M H_2SO_4	223	-	~100 @ 1.48	39.8	288 mV @ 28h @ 10 mA cm^{-2}	[11]
Ru@IrOx	0.05 M H_2SO_4	282	43.32	644.8 @ 1.56	69.1	282 mV @ 2h @ 10 mA cm^{-2}	[12]
MnRuOx-300	0.5 M H_2SO_4	231	36.4	-	50.8	-	[13]

Table S2. The Pt and Ru ratios and masses of the as-prepared nanoparticles used in OER catalysis.

Samples	Pt : Ru weigh ratio	Pt weight (μg)	Ru weight (μg)
Pt nanocube	100 % : 0 %	30	0
Open Ru 31 nm-branch	40.1 % : 59.9 %	12.0	18.0
Open Ru 52 nm-branch	11.8 % : 88.2 %	3.5	26.5
Pure Ru 28 nm-branch	0 % : 100 %	0	30

Table S3. Comparison of calculated electrochemical surface areas (ECSAs) obtained from CO stripping for Pt nanocubes and branched Ru nanoparticles.

Samples	CO stripping results (cm^2)	ECSA ($m^2\ g^{-1}$)
Pt nanocube	12.69	42.3*
Open Ru 31 nm-branch	10.74	35.8*
Open Ru 52 nm-branch	37.58	125.3*
Pure Ru 28 nm-branch	2.75	9.2*

*: normalized by the total mass of metal catalysts.

References

1. H.-S. Chen, T. M. Benedetti, J. Lian, S. Cheong, P. B. O'Mara, K. O. Sulaiman, C. H. W. Kelly, R. W. J. Scott, J. J. Gooding and R. D. Tilley, Role of the Secondary Metal in Ordered and Disordered Pt–M Intermetallic Nanoparticles: An Example of Pt3Sn Nanocubes for the Electrocatalytic Methanol Oxidation, *ACS Catal.*, 2021, **11**, 2235-2243.
2. L. Gloag, T. M. Benedetti, S. Cheong, C. E. Marjo, J. J. Gooding and R. D. Tilley, Cubic-Core Hexagonal-Branch Mechanism To Synthesize Bimetallic Branched and Faceted Pd-Ru Nanoparticles for Oxygen Evolution Reaction Electrocatalysis, *J. Am. Chem. Soc.*, 2018, **140**, 12760-12764.
3. A. R. Poerwoprajitno, L. Gloag, T. M. Benedetti, S. Cheong, J. Watt, D. L. Huber, J. J. Gooding and R. D. Tilley, Formation of Branched Ruthenium Nanoparticles for Improved Electrocatalysis of Oxygen Evolution Reaction, *Small*, 2019, **15**, 1804577.
4. J. J. L. Humphrey, S. Sadasivan, D. Plana, V. Celorrio, R. A. Tooze and D. J. Fermín, Surface Activation of Pt Nanoparticles Synthesised by "Hot Injection" in the Presence of Oleylamine, *Chem. Eur. J.*, 2015, **21**, 12694-12701.
5. Y. Yao, S. Hu, W. Chen, Z.-Q. Huang, W. Wei, T. Yao, R. Liu, K. Zang, X. Wang, G. Wu, W. Yuan, T. Yuan, B. Zhu, W. Liu, Z. Li, D. He, Z. Xue, Y. Wang, X. Zheng, J. Dong, C.-R. Chang, Y. Chen, X. Hong, J. Luo, S. Wei, W.-X. Li, P. Strasser, Y. Wu and Y. Li, Engineering the electronic structure of single atom Ru sites via compressive strain boosts acidic water oxidation electrocatalysis, *Nat. Catal*, 2019, **2**, 304-313.
6. W. He, X. Tan, Y. Guo, Y. Xiao, H. Cui and C. Wang, Grain-Boundary-Rich RuO2 Porous Nanosheet for Efficient and Stable Acidic Water Oxidation, *Angew. Chem. Int. Ed.*, 2024, **63**, e202405798.
7. Y. J. Kim, A. Lim, J. M. Kim, D. Lim, K. H. Chae, E. N. Cho, H. J. Han, K. U. Jeon, M. Kim, G. H. Lee, G. R. Lee, H. S. Ahn, H. S. Park, H. Kim, J. Y. Kim and Y. S. Jung, Highly efficient oxygen evolution reaction via facile bubble transport realized by three-dimensionally stack-printed catalysts, *Nat. Commun*, 2020, **11**, 4921.
8. J. Zhu, Z. Lyu, Z. Chen, M. Xie, M. Chi, W. Jin and Y. Xia, Facile Synthesis and Characterization of Pd@IrnL (n = 1–4) Core–Shell Nanocubes for Highly Efficient Oxygen Evolution in Acidic Media, *Chem. Mat.*, 2019, **31**, 5867-5875.
9. J. Shan, T. Ling, K. Davey, Y. Zheng and S.-Z. Qiao, Transition-Metal-Doped RuIr Bifunctional Nanocrystals for Overall Water Splitting in Acidic Environments, *Adv. Mater.*, 2019, **31**, 1900510.
10. L. Gloag, T. M. Benedetti, S. Cheong, Y. Li, X.-H. Chan, L.-M. Lacroix, S. L. Y. Chang, R. Arenal, I. Florea, H. Barron, A. S. Barnard, A. M. Henning, C. Zhao, W. Schuhmann, J. J. Gooding and R. D. Tilley, Three-Dimensional Branched and Faceted Gold–Ruthenium Nanoparticles: Using Nanostructure to Improve Stability in Oxygen Evolution Electrocatalysis, *Angew. Chem. Int. Ed.* , 2018, **57**, 10241-10245.
11. D. Chen, T. Liu, P. Wang, J. Zhao, C. Zhang, R. Cheng, W. Li, P. Ji, Z. Pu and S. Mu, Ionothermal Route to Phase-Pure RuB2 Catalysts for Efficient Oxygen Evolution and Water Splitting in Acidic Media, *ACS Energy Lett.*, 2020, **5**, 2909-2915.
12. J. Shan, C. Guo, Y. Zhu, S. Chen, L. Song, M. Jaroniec, Y. Zheng and S.-Z. Qiao, Charge-Redistribution-Enhanced Nanocrystalline Ru@IrOx Electrocatalysts for Oxygen Evolution in Acidic Media, *Chem*, 2019, **5**, 445-459.
13. J. Zhang, L. Xu, X. Yang, S. Guo, Y. Zhang, Y. Zhao, G. Wu and G. Li, Amorphous MnRuOx Containing Microcrystalline for Enhanced Acidic Oxygen-Evolution Activity and Stability, *Angew. Chem., Int. Ed.*, 2024, **63**, e202405641.
